# “About Navigating Chaos”: Latin American and Caribbean Mental Health Workers’ Personal Impact Due to SARS-CoV-2 in the First Hundred Days

**DOI:** 10.3389/ijph.2022.1604359

**Published:** 2022-09-06

**Authors:** Martin Agrest, Melina Rosales, Marina Fernández, Tanvi Kankan, Andrés Matkovich, Alberto Velzi-Díaz, Sara Ardila-Gómez

**Affiliations:** ^1^ Proyecto Suma, Asistencia y Rehabilitación en Salud Mental, Buenos Aires, Argentina; ^2^ Instituto de Investigaciones, Facultad de Psicología, Universidad de Buenos Aires, Buenos Aires, Argentina; ^3^ Teachers College, Columbia University, New York, NY, United States; ^4^ Facultad de Psicología, Universidad Nacional de Rosario, Santa Fe, Argentina

**Keywords:** occupational stress, COVID- 19, mental health and wellbeing, mental health services, Latin America

## Abstract

**Objectives:** The personal impact of COVID-19 on mental health care workers (MHWs) has received scarce attention despite their work addressing the emotional wellbeing of those affected by the pandemic. This study aims to analyze Latin American and Caribbean’s MHWs’ subjective impact in connection to working during the initial times of the pandemic.

**Methods:** One hundred and fifty-five persons (*n* = 155) from seventeen countries were contacted in May–June 2020 through a snowball approach. Complementary methodological strategies of analysis used for data triangulation included content analysis, thematic analysis, and interpretative phenomenological analysis.

**Results:** Participants reported feelings of fear, anxiety, anguish, and fatigue. Milder negative impacts (e.g., uncertainty, concern), and complex feelings (e.g., ambivalence) were also frequent. One third of participants acknowledged their capacity to learn from this situation and/or experience satisfaction.

**Conclusion:** Mental health of MHWs in Latin America and the Caribbean were under severe strain and the ongoing mental health reforms at risk during the pandemic’s beginning. More research and additional care may be needed to offer support to those involved in caring for the wellbeing of others.

## Introduction

The SARS-CoV-2 pandemic has affected most people around the globe, despite country and personal differences. Older adults [1], persons with certain comorbidities [2] and intensive care health care workers [3] are vulnerable populations acknowledged as severely impacted. Still others, including minority groups, refugees, and mental health workers (MHWs), have been relatively overlooked. MHWs were called to provide emotional and crisis support to individuals including other health workers, while caring for people more isolated than ever before [4, 5]. MHWs had to cope with sudden transformations amidst two preceding complex situations: 1) mental health services continued to be historically devalued amongst health services [6], and 2) a gradual transition from psychiatric hospital-based to community-based mental health care in the region was ongoing when SARS-CoV-2 made its sudden appearance [4].

Under these circumstances a research consortium in Latin America, which conducted a similar study in Argentina [7], interviewed MHWs from 17 countries in Latin America and the Caribbean (LAC) to investigate their personal impact associated with working in mental health services during the first 100 days of the SARS-CoV-2 pandemic.

### The Onset of the SARS-CoV-2 Pandemic in Latin America and the Caribbean

The pandemic hit the region particularly hard [8] after an initial delay [9]. Large and densely populated countries in the region (e.g., Brazil, México, Colombia) were the most affected (see Table 1). Less populated countries (e.g., Uruguay, Costa Rica, El Salvador) ranked among the least affected in the initial stages of the pandemic. According to national official COVID-19 statistics, Venezuela, despite having a middle range population, was the least affected.

**TABLE 1:** SARS-CoV-2 official statistics of detected cases, deaths and deaths per 100,000 inhabitants by country. “About Navigating Chaos”: Latin American and Caribbean Mental Health Workers’ Personal Impact Due to SARS-CoV-2 in the First Hundred Days, 17 countries in Latin America and the Caribbean, 2020.

Country	First detected case date	Number of deaths by June 13th	Number of deaths per 100,000 inhab. By June 13th	Inhabitants (2020)
Bolivia	10th March	716	6.13	11,673,021
Brazil	26th February	42,791	20.13	212,559,417
Chile	3rd March	3,507	18.34	19,116,201
Colombia	6th March	1,592	3.13	50,882,891
Costa Rica	5th March	12	0.23	5,094,118
Cuba	11th March	84	0.74	11,326,616
Ecuador	29th February	6,051	34.29	17,643,054
El Salvador	19th March	72	1.11	6,486,205
Guatemala	13th March	351	1.95	17,915,568
Honduras	12th March	306	3.09	9,904,607
Mexico	28th February	27,763	21.53	128,932,753
Nicaragua	19th March	55	0.83	6,624,554
Panama	10th March	429	9.94	4,314,767
Paraguay	7th March	11	0.15	7,132,538
Peru	6th March	34,464	104.53	32,971,854
Uruguay	13th March	23	0.66	3,473,730
Venezuela	13th March	24	0.08	28,435,950

Source: Elaborated by the authors based on https://www.worldometers.info/coronavirus/#countries

### SARS-CoV-2 Pandemic and Mental Health

Psychological impacts have been extensively described in connection to isolation and the life-threatening situations associated to COVID-19 [10–12], including symptoms of post-traumatic stress, depression, anxiety, irritability, insomnia, anger, and emotional exhaustion. MHWs providing care for these might need to support their health care colleagues dealing with the difficulties of deciding who to help and who to let die [13]. These responsibilities are compounded by the potentially increased workload in meeting the needs of mental health service users as the latter would be more vulnerable if infected by SARS-CoV-2 because of frequent comorbidities [14, 15]. Despite the increased demand on them, MHWs have not been much targeted for help or research.

Unlike other forms of health care, remote work is feasible for most MHWs and clients, potentially allowing treatments to continue. Telepsychiatry experienced an unexpected boost [16, 17] and has been celebrated as a promising approach to mental health problems globally [18–20]. However, group activities had severe limitations transitioning to virtuality, individuals committed to inpatient units got increasingly isolated, people with no access to technology were segregated, and remote forms of care posed significant challenges for both workers and patients [21, 22].

The personal impact of the challenges MHWs faced is unknown. In this study, as part of a larger study conducted to understand the impact of SARS-CoV-2 in the provision of mental health services in LAC, we have addressed the gap by approaching MHWs to gauge their subjective impact in connection to working during the initial times of the pandemic.

## Methods

### Participants and Procedures

An exploratory study was conducted with MHWs (*n* = 178) from different types of mental health services (e.g., inpatient units, emergency, primary, day hospital and ambulatory care) from 17 countries in LAC between 8th May and 30thJune 2020. Participants were asked about their emotional impact due to SARS-CoV-2 in connection to their work among other questions regarding their work.

A snowball approach was adopted after contacting (by email/WhatsApp message) key MHWs based on personal contacts. Inclusion criteria was defined as any MHW from public or private mental health services with at least 5 years of experience as provider or manager. Participants were invited for a virtual interview as a first preference and offered to complete an online questionnaire as an alternative. The interview guide was identical to the questionnaire. The interview guide comprised: demographic data, type of service and role where the participant worked, pandemic’s effect on service and their personal impact in connection to their work during the pandemic. Data collection spanned less than 2 months to simultaneously capture the initial situation in all LAC countries. No pilot study was implemented. All participants were interviewed or surveyed in Spanish. Brazilian participants could opt to respond in Portuguese, English or Spanish; non-Spanish answers were translated and coded in Spanish. Codes and selected interview excerpts were translated into English and back translated into Spanish to secure the adequacy of translation. The number of participants was decided either when saturation of information was reached or when participants suggested by other participants were not available and the chain ended.

The study was approved by the Commission for the evaluation of responsible research behavior at the Facultad de Psicología (Universidad de Buenos Aires) (Ref. 10/20-2020). All interviewed participants provided consent for their data to be anonymously used in the research.

### Data Analysis

Four researchers (MA, MF, MR, and SAG) were involved in data analysis, conducted in Spanish. Three methodological strategies were used for triangulation: 1) First, answers were independently read and inductively coded by two members of the research team, until they reached consensus on final coding. Codes were translated into English by a bilingual translator with a mental health background. Word clouds with these codes were created for the total answers, by type of mental health service and by community circulation of the virus (i.e., higher or lower infection rate per 100,000 inhabitants) (See Supplementary Material S1). Frequency of the codes and distribution among countries were further calculated. 2) Secondly, by thematically analyzing the responses [23] they were assigned to four types of personal impact (i.e., negative, positive, neutral and mixed). Responses could be assigned to more than one type. Frequencies were also calculated for the types of personal impact (See Supplementary Material S2). 3) Thirdly, an Interpretative Phenomenological Analysis [24] was performed for answers assigned to each of the four groups; subthemes were defined.

## Results

### Number and Distribution of Participants

Participants from 17 countries (*n* = 155) answered the specific question about their emotional impact during the first 100 days of the pandemic (response rate = 87.1%). MHWs from different types of mental health services provided feedback on their emotional impact though these were not equally distributed. Seven countries (i.e., Brazil, Chile, Colombia, Bolivia, México, Ecuador and Perú) accounted for 68% of all answers, while some countries (e.g., Panamá, Cuba, Venezuela, and Nicaragua) provided scant responses (See Table 2).

**TABLE 2:** Number of participants by country and service type. “About Navigating Chaos”: Latin American and Caribbean Mental Health Workers’ Personal Impact Due to SARS-CoV-2 in the First Hundred Days, 17 countries in Latin America and the Caribbean, 2020.

	Service type	Inpatient units (psychiatric hospital)	Inpatient MH units (general hospital)	Rehabilitation services	Primary care	Emergency care	Ambulatory care	Other MH services	Total	
**Country**		**N**	**N**	**N**	**N**	**N**	**N**	**N**	**N**	**(%)**
Bolivia		6	0	0	0	0	9	0	15	(9.7)
Brazil		0	0	5	8	1	2	3	19	(12.3)
Chile		2	3	4	6	0	2	2	19	(12.3)
Colombia		4	3	3	2	2	3	1	18	(11.6)
Costa Rica		1	1	0	2	0	0	1	5	(3.2)
Cuba		0	0	1	2	0	0	1	4	(2.6)
Ecuador		0	3	1	2	0	4	1	11	(7.1)
El Salvador		1	0	0	2	0	2	0	5	(3.2)
Guatemala		2	1	1	0	0	1	0	5	(3.2)
Honduras		2	0	1	1	1	3	0	8	(5.2)
Mexico		2	0	3	3	0	0	5	13	(8.4)
Nicaragua		2	0	0	1	0	1	0	4	(2.6)
Panama		1	0	0	0	0	0	0	1	(0.7)
Paraguay		1	0	2	1	0	2	2	8	(5.2)
Peru		1	0	2	5	0	2	0	10	(6.5)
Uruguay		1	0	3	2	0	0	0	6	(3.9)
Venezuela		2	0	0	0	0	2	0	4	(2.6)
Total	N (%)	28 (18.0%)	11 (7.1%)	26 (7.1%)	37 (23.9%)	4 (2.6%)	33 (21.3%)	16 (10.3%)	155	(100.0) (100.0%)

Each participant received 2.7 codes on average (range = 1–12). However, participants from Cuba and Venezuela had shorter answers and received fewer codes (1.5 and 1, respectively on average), thus having less weight on a general appreciation of the pandemic’s impact on LAC MHWs.

### Types of Emotional Impact

A negative impact was mentioned by 84.5% of participants (*n* = 131) from every country included. Fear (including fear of contagion and infecting others) was mentioned by 30 participants from 13 countries. Exhaustion, overload, and fatigue was mentioned by 31 participants from 11 countries. Stress (including tension, feeling nervous, demanded, and pressed) was mentioned by 26 participants from 11 countries. Feelings of impotence were reported by 14 participants from nine countries.

A positive impact (including feeling motivated, gratified, satisfaction, and happiness) was reported by 16.8% of participants (*n* = 26). Other participants reported the changes due to the pandemic as neutral or having been able to neutralize (including being adapted, careful, and learning how to cope with the pandemic), accounting for 31.6% of participants (*n* = 49) with any positive impact.

Mixed emotions, including being ambivalent, cautious, challenged, and in consideration of risks, was reported by 27.7% of participants (*n* = 43).

Different negative, positive, neutral, and mixed impacts resulting from Interpretative Phenomenological Analysis are depicted below.

### Negative Impact: Anxiety and Fear

Several participants commented on their anxiety and fear. A Colombian participant spoke of his fear of infecting others:

Right now, I’m really scared to death. I feel it [the risk of infection] is closer because now the [strict] quarantine is finishing (…) I feel anguish, not so much for my own wellbeing but due to the possibility of infecting people during home visits and [IC] units I’m working at … Fear is more about infecting others who are at risk than my own (Co #2)

Similarly, others said: “[The pandemic] I think has favored working soaked with fears, doubts, and anxiety on how, what, and when to do things” (Br #21). Being more careful than ever before greatly contributed to constant anxiety and fears of harming people while trying to help them.

### Negative Impact: Economic Problems

Several participants from Colombia mentioned the economic impact of the pandemic on their income along with other problems. “*I spend more time at home, I feel less useful, remote work is more energy consuming and I have visual fatigue. My earnings have gone down by 30%*” (Co #4). Another participant said: “the pandemic has had *an economic impact*. The uncertainty has brought me anxiety and the administrative work has increased” (Co #11). Another psychiatrist from Colombia mentioned that his “*workload as physician reduced, but the administrative work increased* (...) It implied substantial changes at a monetary level” (Co #10).

These comments were not restricted to Colombian participants. One participant from Ecuador said that “[it] certainly affected [me] financially” (Ec #1). A Chilean participant commented on “the fear of losing your job and the fear of economic bankruptcy” (Ch #11).

### Negative Impact: Overload and Exhaustion

Working with constant uncertainty, anxiety and fears, caused exhaustion and feelings of overload.

It is quite uncertain how things are going to be tomorrow, or until when. Not knowing if you are doing the right thing or imposing risks to patients or to others, feeling a greater pressure to have the [mental health] services empty and ready, and *this means a substantial overload* (...) You don’t respect your schedule and *you don’t easily understand that you are getting more tired* (Ch #10).

Another participant said that “a greater number of new cases to treat has determined a mental and emotional exhaustion, especially without the necessary tools for those who contain others to feel contained” (Ec #13).

A Chilean psychologist described how this pandemic has affected him despite his experience in working with crisis relief: “I’m more exhausted. Cases are more complex, and you need constant adaptability (...) *I am well trained in catastrophes, particularly oriented to earthquakes. But this is more than navigating uncertainty. This is about navigating chaos*” (Ch #11).

### Negative Impact: Anguish and Tragic Metaphors

Along with negative feelings, war, climate, or other catastrophes were mentioned for comparison, to end up stating that this pandemic was unlike anything they knew. A psychiatrist from El Salvador compared the pandemic with a “monster.”

I have worked for 40 years. *I have never thought I would go through a pandemic like this*. *The first one to be affected was myself* (...) I told my son: “I was in a war, and this will not affect me.” But *then I realized that this has nothing to do with war. This pandemic is a monster,* and you cannot see its head or tail (ES #3).

One participant from Brazil considered that the worst was ahead and said: “we are still in the eye of the storm” (Br #16).

Two participants from Guatemala expressed their emotional pain saying: “I think I was okay at the beginning, but now I’m going down. I feel a great pressure, anguish, and fatigue. I don’t know how I will be able to keep this amount of work.” (Gu #4). “This was a dramatic change (…) Students who had started [working] in January had to stop practicing in March, which generates great anguish in them and myself.” (Gu #2).

### Negative Impact: Feelings of Impotence and Mental Health Being Relegated Adding on to Political Criticisms

Participants repeatedly mentioned discomfort with mental health being disregarded at the policy level. One participant said:

*I’m waiting for this to come to an end to submit my resignation*. The pandemic allowed me to realize that the institution [where I work] is not helping. I’ve been doing this for 23 years. But now I see [the institution] does not care about people’s mental health nor does it allow [anybody] to work (Bo #15).

Some participants mentioned that they were “sent home” to be protected from getting infected if they belonged to a vulnerable group. However, this carefully taken decision also gave way to impotence for not being able to stay in touch with those in need. A participant from Uruguay said this had been “horrible” for her not knowing if her patients were able receive any food in replacement to what they received in the day care center that had been closed.

Participants from Chile and Nicaragua mentioned the prior socio-political context, explicitly connecting the current situation with a wider and global determination that went beyond mental health.

“[This is] tremendous (...) *Services we are providing - I don’t think they are enough, and [they] do not have the quality I would prefer … It strikes me that resources are always limited*. My impression is that the State is saying: ‘you can only do up to here.” (Ch #4).

Similarly, a psychiatrist from Nicaragua said:

This affects me. I am frustrated to see the government stance (...) I feel powerless (...) If mental health had received greater attention [from the government] I would have been back to the hospital as a volunteer (...) On a personal level, *I feel impotent, frustrated because the government hasn’t given [covid-19] enough attention and even less to mental health* (Ni #1).

In line with this, another participant reported feeling: “*abandoned by the government*, with decisions that are taken just ‘for the screens’ [to publish them, while] there is no real concern for our lives, for our protection or true value given to what we are doing” (Pe #1). In Venezuela, one participant considered that “COVID-19 has received all the attention and *‘mental health patients’ have been abandoned*” (Ve #4).

### Positive Impact: Satisfaction

Reports of positive feelings were few and rare without being connected to difficult experiences. There was little elaboration from most who did mention positive experiences exclusively; for instance, one participant merely said the experience had been “gratifying” (Br #23).

Another participant showed satisfaction and hope for how the pandemic could enhance an incipient and important community strategy:

*This [pandemic] comes to reinforce the direction we have always wanted to take*, and now there is a greater commitment and value of our work (...) We have a lot of expectations because the covid pandemic will go and I hope this energy [we are having] can be sustainable (Ch #21)

A psychologist from Costa Rica (CR #5) considered that “at the beginning [the impact] was favorable, mostly because she could stay comfortably at home, protecting herself and her family.” A psychiatrist from Ecuador (Ec #7) said that “it was a brilliant opportunity for [certain community] services to emerge.”

In fact, more frequently positive feelings were balanced with complex or negative experiences. One participant from Chile said: “*I’m learning … happy for all we have been able to do so far*” (Ch #9). However, he also mentioned to have “spent a night with almost no sleep and considered the worst-case scenario. (...) *This has been really exhausting*.”

In Honduras, one participant began by talking about feeling “impotent, frustrated, and angry because of circumstances of careless misconduct” and then added: “When I see that the strategy is successful, that people are doing better, I feel energized … *I’m so satisfied! I love mental health*. I’m in love with it” (Ho #1).

### Neutralizing Negative Impact Through Adaptation and Learning

Some participants mentioned that they were able to adapt to the situation and neutralize the pandemic’s effects. A participant from Honduras said that he had been able to “understand and adapt” (Ho #7). A psychiatrist from Paraguay stated they “did not envision the impact [covid-19] would have in the country. However, as other countries in the region started to suffer this impact, the [health] ministry got in touch with us, and I think the measures taken so far have been successful (...) We have implemented all possible biosecurity measures.”

Participants shared how finding ways to be helpful facilitated coping. A participant from Uruguay said that “at a personal level I have been proactive, searching for ways to avoid the arising difficulties and staying close to the [patients]” (Ur #3). A psychologist from Honduras mentioned: “At the beginning I felt uncomfortable (…) I felt guilty for not being able to receive the people who I was sure were needing help. When [consultation over] the phone was available at the hospital I was able to feel somewhat in peace” (Ho #2).

Some participants were able to transform initial fears into an asset.

I have been deeply impacted, especially at the beginning. *I was scared*. I was affected at a professional level and at a familial level (...) *I was very worried about bringing the virus home*. Then, I learnt about biosecurity. I’m currently offering lectures and making presentations on emotional coping and biosecurity (...) *I feel confident now* (...) *I know the topic, I know myself, and I trust in God* (Ho #8).

Others also commented on their capacity to learn: *“I’ve really felt scared* (...) *We’ve been forced to learn* and tighten hand washing (...) The pandemic has taught us two things: to live better and to care for our health. It is not that bad” (Pe #11).

### Mixed and Complex Impact: Taking the Bitter With the Sweet

Several participants exuded more than just negative or positive impacts, alluding to complex ways of living the pandemic. A Chilean participant said:

*It is a challenge and a responsibility*. I feel a lot of pressure (...) With other colleagues we feel like we have prepared for critical situations like this one and it is time to just do it. We see how the system is working with adequate connections and it is somehow satisfactory. I also feel uncertain. (Ch #5).

Some participants, such as the following professional from Colombia, showed an interesting mix of emotions.

At a personal level *this has been a real challenge for me*, a challenge full of anxiety and worries. At the beginning, [we had] a lot of stress in connection with an increase in the amount of work (...) *It was a roller coaster of emotions* (Co #13).

A MHW from Guatemala said: “Work has increased a lot. And being female, it is even harder. If I was a male doctor, I would be considered a hero in this context. It is tiring (...) I hope a new and better way of living and to relate to each other emerges from this” (Gu #3). Another participant from Nicaragua began saying: “My initial reaction was being in shock. I was extremely afraid because I am 60 years old. On the other hand, it was very nice to see the solidarity among those who were close to the clinic” (Ni #4).

## Discussion

The most significant aspect pointed out by participants was how things changed for them in initial months, and how things kept changing. The temporal dimension (i.e., how MHWs felt affected in different ways as the pandemic evolved) was repeatedly underscored. Adapting to and learning from these changes appeared feasible for some MHWs but took a toll on many.

### Prevalent Though Not Unanimous Negative Emotional Experience

Expectedly, different negative emotional experiences were mentioned by participants. Concern and discomfort accounted for mild and less frequent negative reactions (feeling unprepared; experiencing uncertainty and unpredictability). Fear, anxiety, stress, anguish, and fatigue denoted a more severe and common negative impact across almost every country.

Some participants talked about feeling depressed, having sleep disturbances, experiencing anger, frustration, isolation, or guilt, and being disappointed in acknowledgement of their vulnerability during the pandemic. More extreme feelings (e.g., “chaos”, being in “shock,” the situation being “horrible”) were common even as MHWs were expected to provide solace to other health professionals and service users. Similar patterns were later confirmed in other regions through anxiety, depression, and burnout scales [25, 26]. However, metaphors frequently used by MHWs in LAC conveyed vivid desperation and, for some, a tragic experience of the pandemic.

Both female and male participants commented on their difficulties balancing work and familial responsibilities. Regardless of working remotely or in health facilities which may threaten their families by bringing the virus to their home, participants expressed their fears and concerns towards their family. This latter concern (“the risk family and friends may be infected through me”) was reported by Johnson et al [27] for as many as 47% of nurses participating in a United Kingdom-based study. Unsurprisingly, MHWs in LAC, where family bonds are typically intense and prioritized [28], highlighted their fear of infecting their families.

### Silver Lining: Global Perception, Positive Perspective on Commitment and Responsibility

One in six participants commented on their satisfaction, joy, happiness, optimism, ease and/or motivation. An additional 15% of participants acknowledged their capacity to learn from this situation and eventually adapt to it. In Italy and the United Kingdom, two studies capturing data later in the pandemic showed higher percentages [29, 30]. In Italy, 59.6% of participants stated that they found some positives in the COVID-19 experience [29] and, in the United Kingdom, resilience was reported for as much as 70% of participants [30]. The timing of our response collection at the onset of the pandemic, may be a factor in explaining why a lower percentage of participants included positive aspects of their experience. Most participants expressed negative impacts along with the positives; mixed emotions were more common.

Distance from frontline, greater seniority and professional fulfillment, have been reported in other studies as protective factors for burn-out during the pandemic [25, 31], although seniority had been pointed as a negative factor in pre-pandemic studies [32]. Our study could not offer support in either direction. However, participants that framed their experience as a “challenge” and highlighted their “responsibility,” expressed a fatigue similar to others but a greater optimism and benevolence regarding services being provided. Focusing on individuals able to receive adequate services, may have protected participants from more negative feelings. Despite the pandemic, some participants exuded a significant sense of control over the situation and thus alluded to a more positive impact.

### Mental Health at the Crossroad of Unfulfilled Transformations and Political Neglect

Participants from all 17 countries mentioned institutional limitations and challenges connected to government’s decisions. Personal impact was associated with what mental health care services could or could not do. Positively, collaborative work, support among colleagues, and greater networking were mentioned by participants from a few countries (e.g., Bolivia, Brazil, Paraguay, and Uruguay) as protective factors. This finding aligns with a later study among United Kingdom MHWs in which support and information from colleagues was rated as very or extremely important by 64% of respondents [27].

However, most comments referring to the institutional (or system) level implied negative aspects. “Lacking institutional support,” “disappointment with the government,” “invisibility of mental health,” and “increased working complexities,” involved participants from every country. With other forms of discomfort, mental health shortcomings and other restrictions frequently highlighted within the field (e.g., budget-related), contributed to the negative impact reported by participants. Amidst psychiatric reforms in the region, such perspectives would be indicative of an alarming situation that can hopefully prompt envisioned reforms.

### Strengths, Limitations, and Future Directions

Despite the intention to contact MHWs from all LAC countries, and reaching 155 MHWs, several limitations must be highlighted: 1) Some countries are underrepresented in the sample (e.g., Panama, Nicaragua); 2) The snowball approach limits generalizability of results; 3) Participants from Brazil, Cuba and Venezuela self-administered the questionnaire more often than participants from other countries, allowing fewer elaborations and codes; 4) Not every type of services were equally reached 5) Unreliable reporting of statistics (e.g., Nicaragua and Venezuela); cast doubt on correlation between infection spread and personal impact by country; 6) Most countries included in the sample have no representatives in the research team; 7) Comparisons with other regions and at different stages of the pandemic should be made with caution since different methodologies were employed (i.e., spontaneous reports vs queried answers).

However, the number of countries and the variety of participants’ background and experience included in the sample are significant strengths. To the best of our knowledge, this is the first report based on interviews with MHWs from multiple LAC countries gauging their personal impact in connection to working during the pandemic.

Studies on LAC MHWs’ situation at later stages of the pandemic that include multiple countries are still pending. The situation depicted by the participants from our study can provide a benchmark for such future studies. Broadening our understanding, and acting upon it, would be mandatory to care for those who care for the carers and for vulnerable populations, and to secure the mental health reform in the region.

## Supplementary Material

Supplementary Figure S1 Word clouds by type of mental health service and by the community circulation of virus. “About Navigating Chaos”: Latin American and Caribbean Mental Health Workers’ Personal Impact Due to SARS-CoV-2 in the First Hundred Days. 17 Latin American and the Caribbean countries. 2020.

Supplementary Figure S2 Codes’ frequency for the 155 participants. “About Navigating Chaos”: Latin American and Caribbean Mental Health Workers’ Personal Impact Due to SARS-CoV-2 in the First Hundred Days. 17 Latin American and the Caribbean countries. 2020.
