# Supplementary material for: “About Navigating Chaos”: Latin American and Caribbean Mental Health Workers’ Personal Impact Due to SARS-CoV-2 in the First Hundred Days
Source: Int J Public Health. 2022 Sep 6;67:1604359. doi: 10.3389/ijph.2022.1604359 (PMC9485453; doi:10.3389/ijph.2022.1604359)
Supplement: Supplementary file 1 [file Image2.pdf]

	Countries N	Countries %	Participants N	Participants %	Participants
Negative impact					
Stress	9	52,9%	16	10,3%	Bo2 Bo8 Bo9 Bo10 Br10 Br24 Ch7 Co8 Co9 Co13 Ec12 Me12 Pa1 Py4 Py7 Pe4
Tension	5	29,4%	7	4,5%	Br3 Br11 Ch5 Co9 Co13 Cr3 Ec1
Demanded/ Pressed/ Pressure	6	35,3%	6	3,9%	Bo8 Br7 Ch10 Cr4 Ec8 Gu4
Fear	11	64,7%	22	14,2%	Bo3 Bo5 Br15 Br20 Br21 Ch7 Ch11 Co2 Co5 Co13 Ec4 Ec13 Es1 Es4 Ho1 Ho8 Me4 Me5 Me7 Pa1 Py11 Pe11
Fear of contagion	7	41,2%	8	5,2%	Bo6 Ch3 Co9 Co18 Cr1 Ec1 Ho8 Ni4
Fear of infecting	1	5,9%	1	0,6%	Ch22
Panic	1	5,9%	1	0,6%	Py11
Anger/ Angry/ Upset/ nervous	3	17,6%	3	1,9%	Br7 Ch3 Ho1
Sleep disturbances / Insomnia	3	17,6%	3	1,9%	Bo2 Br11 Ch7
Acceleration/ Less time/ rush	2	11,8%	2	1,3%	Bo8 Ni5
Anxiety	6	35,3%	12	7,7%	Bo3 Bo4 Bo13 Br11 Ch14 Ch16 Co1 Co3 Co11 Co13 Ho3 Py7
Uncertainty	6	35,3%	11	7,1%	Bo5 Ch5 Ch10 Ch11 Ch14 Ch15 Co7 Co11 Cr1 Cu6 Ur2
Lack of preparation/ Novelty/ Unpredictibility	3	17,6%	5	3,2%	Br16 Me1 Me4 Me16 Pa1
Anguish	7	41,2%	11	7,1%	Bo8 Br19 Br21 Ch7 Ch14 Ch16 Co2 Ec5 Gu2 Gu4 Ur5
Frustration/ Frustrated/ Disappointed	8	47,1%	10	6,5%	Br6 Ch15 Ch20 Co18 Ec4 Ec11 Es2 Ho1 Me16 Ni1
Overload / Emotional overload	8	47.1%	12	7,7%	Bo8 Bo10 Br11 Ch10 Ch15 Co15 Cu2 Ec8 Ec11 Ho1 Ho10 Me2
Worn out	4	23,5%	6	3,9%	Ch10 Co15 Co18 Ec4 Me4 Me16
Burden	2	11,8%	2	1,3%	Bo3 Ch15
Exhaustion/ Exhausting/ Exhausted/ Tiring	6	35,3%	9	5,8%	Bo7 Bo15 Br20 Ch9 Ch11 Cr2 Gu3 Gu5 Pe3
Fatigue	4	23,5%	5	3,2%	Ch10 Ch15 Co4 Ec13 Gu4
Denial	2	11,8%	2	1,3%	Bo4 Es3
Depression/ Depressed/ Sad/ Sadness	3	17,6%	3	1,9%	Bo4 Ch22 Co7
Guilty	1	5,9%	1	0,6%	Ho2
Sorrow	2	11,8%	2	1,3%	Ho11 Pe11
Demotivation/ No vision of future	1	5,9%	1	0,6%	Br17 Co1
Less useful	1	5,9%	1	0,6%	Co4
Irritable	1	5,9%	1	0,6%	Bo2
Impotence	9	52,9%	14	9,0%	Bo2 Br14 Br15 Ch15 Ch20 Co7 Co9 Ec5 Es2 Es4 Ho1 Ho11 Ni1 Ur1
Disarranged / Unstable	2	11,8%	2	1,3%	Co17 Es5
Suffering	2	11,8%	2	1,3%	Co13 Ec10
Concern/ Restlessness	7	41,2%	12	7,7%	Bo1 Bo5 Bo12 Bo14 Co13 Ec10 Gu4 Ho8 Py4 Pe7 Pe10 Pe13
Uncomfortable/ Discomfort (using PPE)	3	17,6%	3	1,9%	Ch11 Co18 Ho2
No support (in general)	1	5,9%	1	0,6%	Ch22
Lost/ Disoriented/ Vulnerable	3	17,6%	3	1,9%	Ch18 Cr1 Gu2
Loneliness/ Isolated/ Abandoned	3	17,6%	3	1,9%	Ch14 Es4 Pe1
Economic impact/ lower income	3	17,6%	7	4,5%	Co4 Co11 Co12 Co17 Co18 Cr1 Ec11
Parenting/ Childcare problems	3	17,6%	3	1,9%	Br11 Ch22 Ho8
Tremendous	1	5,9%	1	0,6%	Ch4
Horrible	1	5,9%	1	0,6%	Ur5
In shock	1	5,9%	1	0,6%	Ni4
Chaos	2	11,8%	2	1,3%	Bo3 Ch11
Difficulty working as a team	1	5,9%	1	0,6%	Br20
Lack of institutional support/ Not valued	4	23,5%	5	3,2%	Bo8 Bo15 Ch22 Es2 Pe1
Invisibilized (MH not visible)	1	5,9%	1	0,6%	Ve4
Disappointement (with State)	1	5,9%	1	0,6%	Br6
Resourceless (Lack of information/ supplies)	3	17,6%	3	1,9%	Ch19 Cr1 Py6
Limitations (unspecified)	11	64,7%	17	11,0%	Br14 Br19 Ch4 Ch6 Co18 Ec12 Ec13 Gu1 Ho1 Ho2 Ho11 Me12 Me16 Ni5 Py12 Pe11 Ur5
Expressing affections/ to contain others	1	5,9%	2	1,3%	Ec1 Ec3
Technological limitations	1	5,9%	1	0,6%	Co18
Missing hospital/ Relocated/ No privacy	1	5,9%	3	1,9%	Co3 Co5 Co6
Conflict/ Job insecurity	2	11,8%	2	1,3%	Bo8 Br5
Less workload/ Inactive	3	17,6%	3	1,9%	Br21 Pe2 Me13
More work/ higher workoad	4	23,5%	4	2,6%	Br24 Ch22 Co11 Me11
Complexity/ Increased work complexity	2	11,8%	2	1,3%	Ch11 Co5
High institutional requirements/ Demands	1	5,9%	1	0,6%	Ch19
Lower efficiency / Administrative problems	2	11,8%	2	1,3%	Co10 Me3
(Negative) emotional impact (unspecified)	3	17,6%	4	2,6%	Co12 Cu5 Es1 Es4
Learning & other positive impacts					
Learning / Reflection	10	58,8%	15	9,7%	Bo5 Br20 Ch9 Ch18 Co13 Ec4 Ec7 Es1 Es3 Es5 Ho1 Ho8 Me2 Pe11 Ur4
Adaptation/ Adaptability/ Training	9	52,9%	14	9,0%	Bo8 Bo13 Ch11 Ch19 Co3 Ec4 Es5 Gu2 Ho5 Ho7 Me5 Me10 Me16 Pe3
Motivated (for creativity)/ Creativity	2	11,8%	2	1,3%	Br20 Co14
Gratifying / Priviledged	2	11,8%	2	1,3%	Br23 Ch11
Happy/ Happiness/ Joy	2	11,8%	3	1,9%	Ch3 Ch9 Ni4
Job appreciation/ Satisfaction	3	17,6%	4	2,6%	Ch5 Ch21 Gu3 Ho1
Optimism/ Hope	3	17,6%	4	2,6%	Ch8 Gu3 Ho3 Ho8
Self-confident	1	5,9%	1	0,6%	Ho8
Opportunity	2	11,8%	2	1,3%	Co8 Ho3
Favorable/ Positive	3	17,6%	3	1,9%	Cr5 Ec2 Me5
Tranquility/ Quiet	2	11,8%	2	1,3%	Ho1 Ni3
Active/ Proactive	2	11,8%	2	1,3%	Ni4 Ur3
Commitment	2	11,8%	2	1,3%	Ch21 Ho1
Challenge	4	23,5%	5	3,2%	Ch5 Co13 Me2 Me14 Ni3
Responsibility/ Time to help others	4	23,5%	6	3,9%	Ch5 Ch9 Co13 Ec4 Me16 Ve2
Security/ Self-care	3	17,6%	4	2,6%	Ch10 Es5 Ho5 Ho8
Collaborative work/ Networking	2	11,8%	2	1,3%	Bo10 Ur5
Importance of co-workers	1	5,9%	1	0,6%	Br5
Support to/ from colleagues	2	11,8%	3	1,9%	Bo3 Bo5 Ch18

Visibilized (Visibility of mental health)	1	5,9%	1	0,6%	Py9
Changing through time & positive evolution	6	35,3%	9	5,8%	Bo3 Bo4 Bo8 Co13 Cu6 Es1 Es3 Ho8 Pa1
No personal impact	1	5,9%	1	0,6%	Ur6
Mixed feelings					
Careful	10	58,8%	15	9,7%	Bo5 Bo14 Br22 Ch10 Co2 Co18 Ec1 Es3 Ho3 Ho8 Ho11 Ni2 Ni3 Py3 Pe11
Caution/ Risk	5	29,4%	5	3,2%	Bo9 Br7 Ch22 Me9 Pe4
Effortful/ Doubtful	2	11,8%	2	1,3%	Bo4 Br21
Patience/ Needing more time	2	11,8%	2	1,3%	Br22 Py5
Swaying emotions	1	5,9%	1	0,6%	Co13
Ambivalence/ Contradictions	2	11,8%	2	1,3%	Br7 Ec4
