# Supplementary material for: “About Navigating Chaos”: Latin American and Caribbean Mental Health Workers’ Personal Impact Due to SARS-CoV-2 in the First Hundred Days
Source: Int J Public Health. 2022 Sep 6;67:1604359. doi: 10.3389/ijph.2022.1604359 (PMC9485453; doi:10.3389/ijph.2022.1604359)

Figure. Word cloud of codes for participants working at General Hospital (N=11)

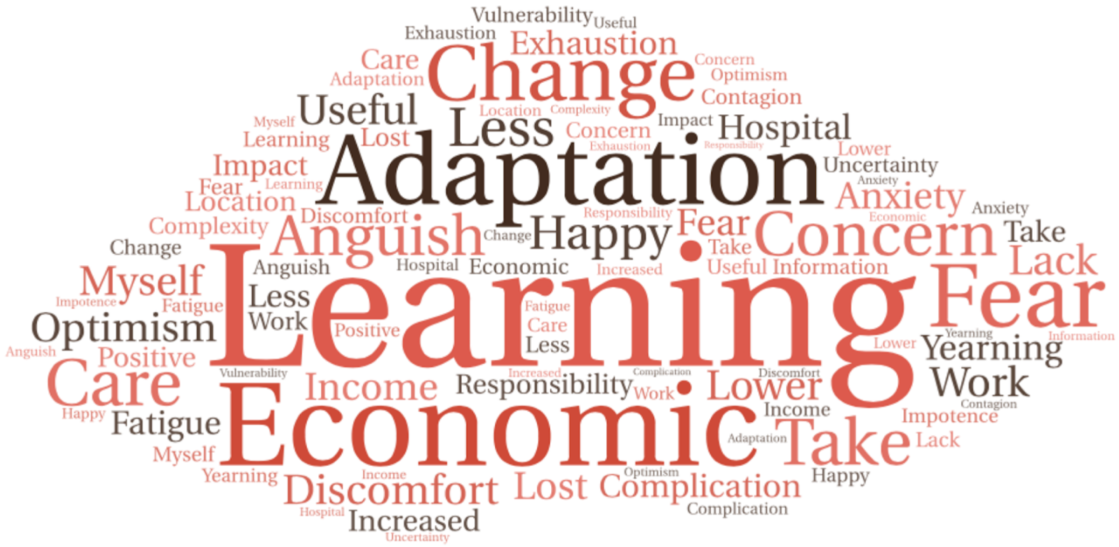

Figure. Word cloud of codes for participants working at Psychiatric hospital (N=28)

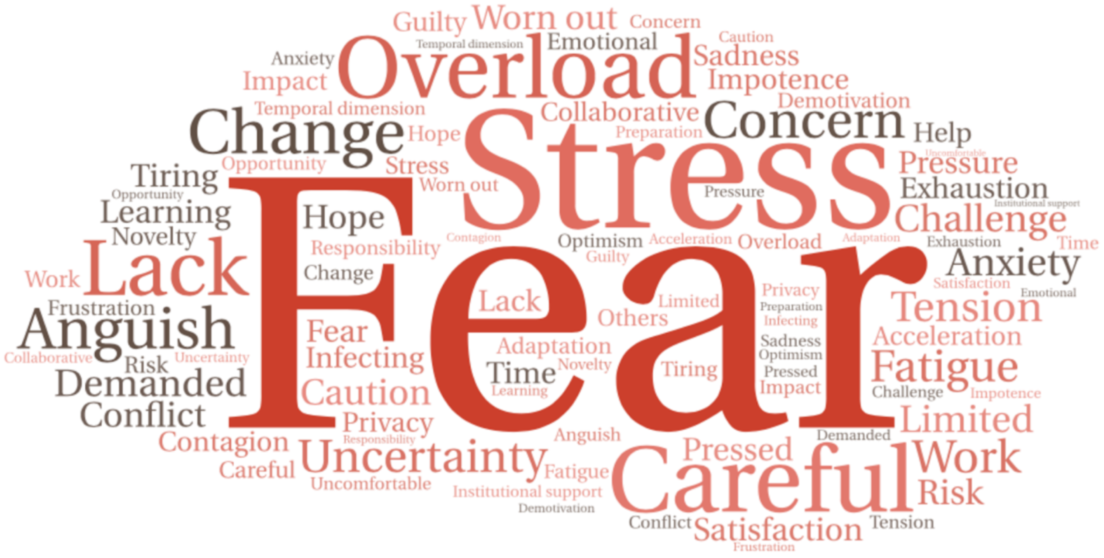

Figure. Word cloud of codes for participants working at Rehabilitation Services (N=26)

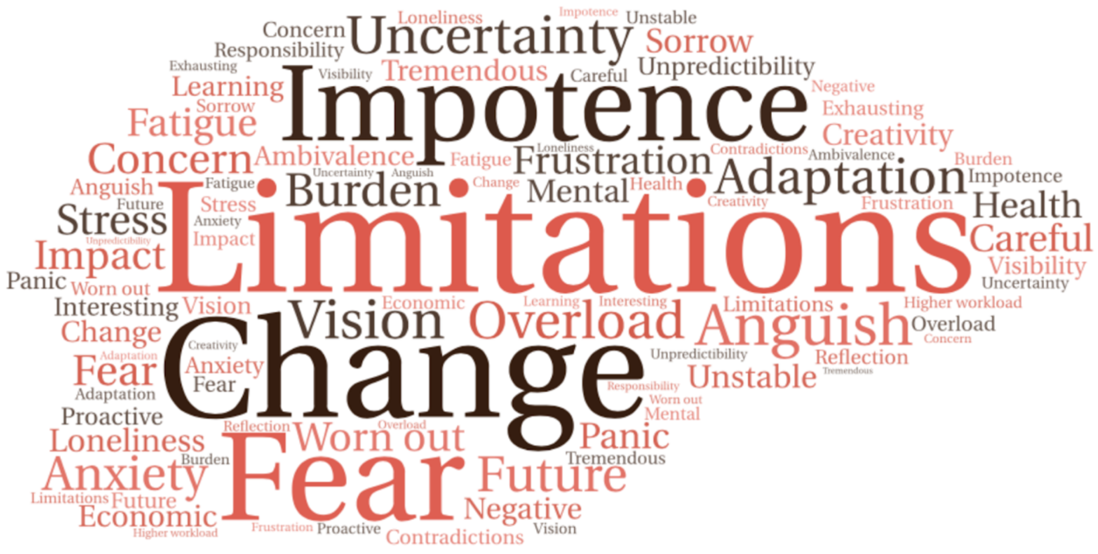

Figure. Word cloud of codes for participants working at Primary Care Services (N= 37)

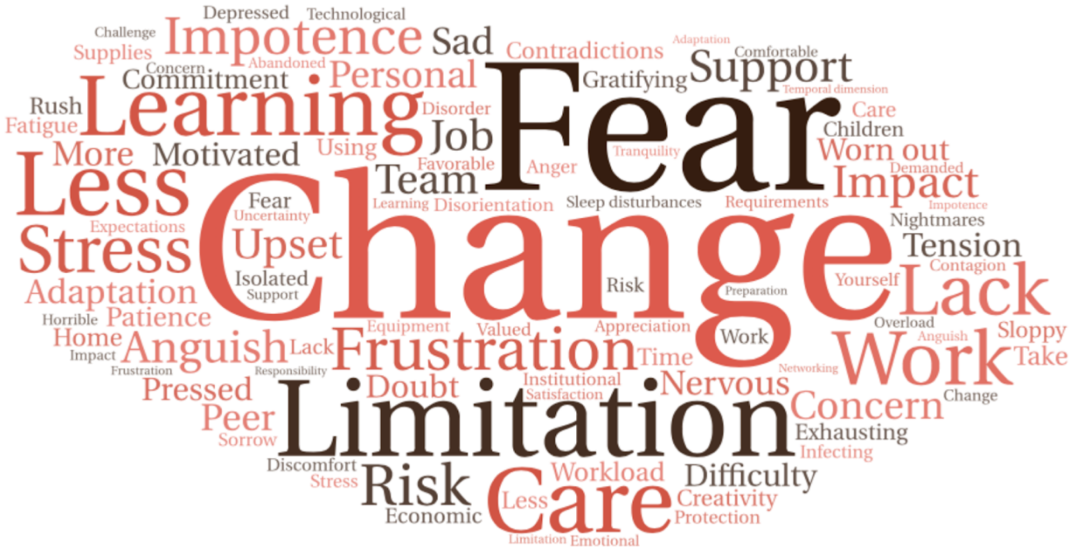

Figure. Word cloud of codes for participants working at higher prevalence countries (Bolivia, Brazil, Colombia, Chile, Ecuador, Honduras, Mexico, Panama, Peru)

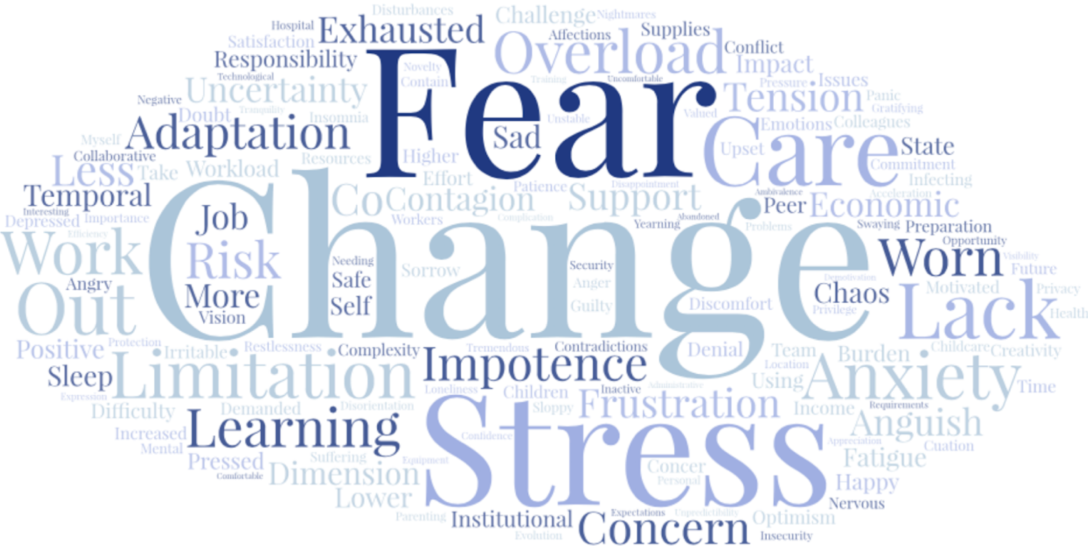

Figure. Word cloud of codes for participants working at lower prevalence countries (Costa Rica, Cuba, El Salvador, Guatemala, Nicaragua, Paraguay, Uruguay, Venezuela)

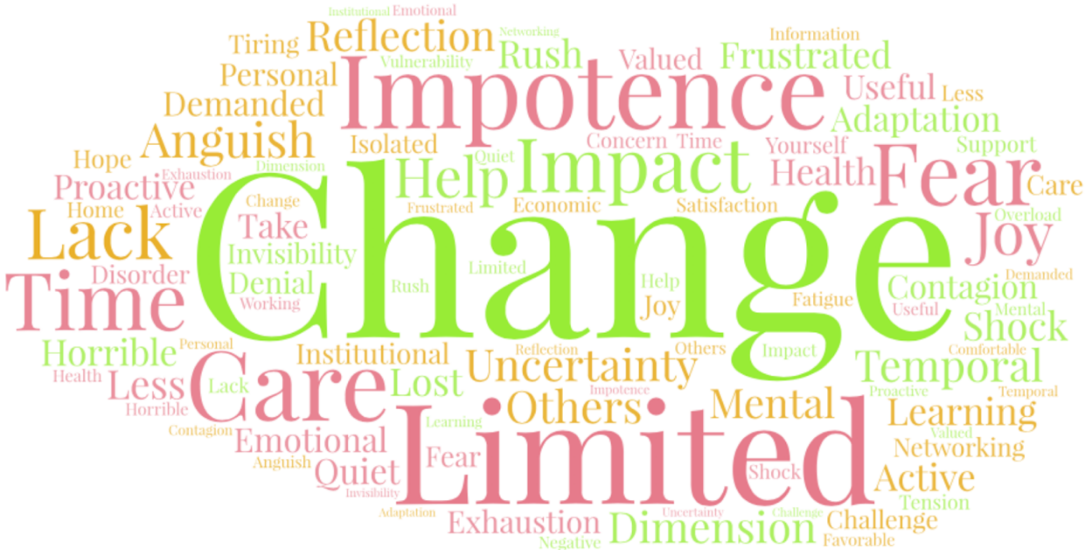

Supplement: Supplementary file 2 [file Image1.pdf]
